# Ollier Disease, Acute Myeloid Leukemia, and Brain Glioma: IDH as the Common Denominator

**DOI:** 10.3390/cancers16183125

**Published:** 2024-09-11

**Authors:** Sergio Corvino, Teresa Somma, Francesco Certo, Giulio Bonomo, Erica Grasso, Felice Esposito, Jacopo Berardinelli, Giuseppe Barbagallo

**Affiliations:** 1 Department of Neurosciences, Reproductive and Odontostomatological Sciences, Neurosurgical Clinic, School of Medicine, Università di Napoli “Federico II”, 80131 Naples, Italy; teresa.somma85@gmail.com (T.S.); felice.esposito@unina.it (F.E.); iacopobe96@gmail.com (J.B.); 2 Department of Neurosciences, Division of Neurosurgery, Policlinico “G. Rodolico-S. Marco”, University Hospital, 95123 Catania, Italy; francesco.certomd@gmail.com (F.C.); dott.giuliobonomo@gmail.com (G.B.); egrasso360@gmail.com (E.G.); gbarbagallo@unict.it (G.B.)

**Keywords:** Ollier disease, brain gliomas, acute myeloid leukemia, enchondromatosis, IDH mutations

## Abstract

**Simple Summary:**

Thanks to the continuous refinement of diagnostic tools, the role of molecular markers analysis has become even more decisive in tumor diagnosis and classification and, accordingly, in the decision-making process of treatment, as emphasized by the most recent WHO Classification of the Tumors of the Central Nervous System (2021), and also by the WHO Classification of Hematolymphoid Tumors (2022). In this setting, the possibility to identify a genetic mutation common to three apparently completely different neoplasms, such as enchondromatosis, acute myeloid leukemia, and brain glioma, and affecting the same patient not only provides a better understanding of the bases of tumorigenesis but also opens the doors to the development of new targeted therapies and experimental treatments which could be effective for apparently different diseases. The present study identified in the IDH1-R132H gene mutation the etiopathogenetic common denominator among Ollier disease, brain glioma, and acute myeloid leukemia coexisting in the same patient.

**Abstract:**

Ollier disease (OD), acute myeloid leukemia (AML), and brain glioma (BG) are three apparently completely different neoplasms in terms of histopathology, clinic, natural history, and management, but they can affect the same patient. This study aimed to identify the common molecular pathways involved in the pathogenesis of all three diseases and discuss their current and potential role as therapeutic targets. A detailed and comprehensive systematic literature review according to PRISMA guidelines on OD patients harboring BG and/or AML was made. In addition, the unique case of a patient affected by all three considered diseases has been added to our case series. Demographic, pathological, treatment, and outcome data were analyzed and discussed, mainly focusing on the molecular findings. Twenty-eight studies reported thirty-three patients affected by OD and BG, and only one study reported one patient with OD and AML, while only our patient harbored all three pathologies. The IDH R132H mutation was the only genetic alteration shared by all three pathologies and was simultaneously detected in enchondromas and brain glioma in 100% (3/3) of OD patients with BG and also in the neoplastic blood cells of the single patient hosting all three diseases. The IDH1-R132H gene mutation is the etiopathogenetic common denominator among three apparently different tumors coexisting in the same patient. The adoption of mutant-specific IDH1 inhibitor molecules could represent a potential panacea for these conditions in the era of targeted therapies. Further studies with larger clinical series are needed to confirm our results and hypothesis.

## 1. Introduction

Ollier disease (OD) is a rare nonhereditary type of dyschondroplasia, belonging to the enchondromatosis, featuring areas of dysplastic cartilage that mainly involve long tubular bones and with asymmetric distribution [1,2], associated with a rate of sarcomatous degeneration of enchondromas ranging from 20 to 50% [1,3,4]. Surgery represents the only curative treatment for primary disease and its complications. OD can be associated with other malignancies, with a prevalence of 53% [5], including mesenchymal tumors such as angiosarcomas, osteosarcomas, and thyroid adenomas, or non-mesenchymal tumors such as leukemia [6], ovarian carcinoma [7], and central nervous system gliomas [8].

Acute myeloid leukemia (AML) is an aggressive malignancy of the white blood cells, characterized by unbridled myeloid blast proliferation and block in differentiation, accounting for symptoms related to ineffective normal hematopoiesis, thus leading to cytopenia and infections including anemia and bleeding [9]. The goal of treatment is to achieve complete remission to reduce the leukemia burden, followed by post-remission therapy administration, represented by chemotherapy and/or allogenic stem cell transplantation [9,10]. The estimated 5-years overall survival has improved from 18% in the year 2000 to 30% currently [11], with the possibility of further improvement in the future thanks to the latest drugs approved and ongoing trials [10].

Brain gliomas (BG) are tumors of the central nervous system arising from glial cells and belonging to the group of *gliomas*, *glioneuronal tumors*, *and neuronal tumors* [12]; they are classified into four grades of malignancy based on molecular parameters, degree of proliferation evaluated through proliferation index, and presence/absence of microvascular proliferation and necrosis. Current guidelines of treatment at initial diagnosis suggest maximal safe tumor resection followed by radio- and chemotherapy based on IDH status and grade [13]. Nevertheless, despite the aggressive treatment, the prognosis remains dismal both for low-grade and high-grade gliomas.

These three apparently very different neoplasms can affect the same patient in various combinations, as reported in the pertinent literature [6,8]. However, most of the current knowledge on the association of OD with synchronous or metachronous BG and/or AML comes from studies belonging to the pre-molecular era [6,8].

Thanks to the continuous and rapid refinement of diagnostic tools [14], and thereby to a better understanding of the genetic bases of tumorigenesis, as well as the impact of the molecular markers on the diagnosis, prognosis, and prediction of response to treatment, nowadays molecular biology has assumed a leading role in the management of neoplastic patients with or without inherited syndromes.

This concept is valid both for brain gliomas, as witnessed by the most recent WHO Classification of Tumors of the Central Nervous System (2021) [12], where molecular findings affect the tumor type and grade, and, accordingly, the diagnosis and management, as well as for acute myeloid leukemia, as witnessed by the updated [15] and the new AML Classification [16], where molecular findings guide the diagnosis, risk stratification, and treatment.

Determining the common molecular pathways and their role in the pathogenesis of the different diseases can aid in the management and development of therapeutic targets. This setting fits our research to define, from a detailed and comprehensive systematic literature review, the main molecular markers shared by apparently very different neoplasms harbored by the same patient, namely Ollier disease, acute myeloid leukemia, and brain glioma, and their potential therapeutic roles. In addition, to the best of our knowledge, we added the first case in the literature of a patient with OD also affected by AML and BG.

## 2. Materials and Methods

**Figure 1:**
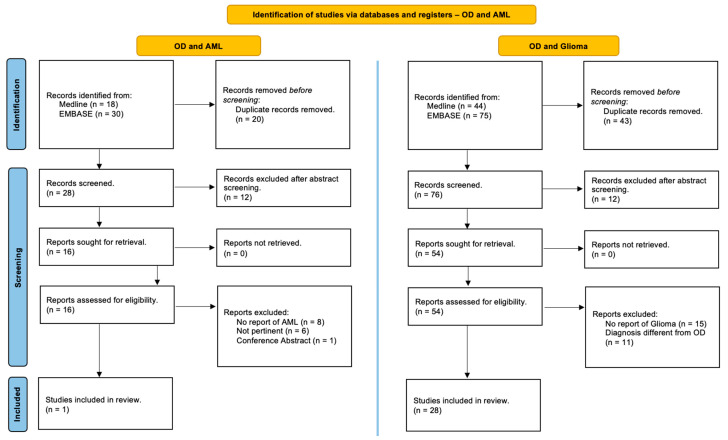
PRISMA (Preferred Reporting Items for Systematic Reviews and Meta-Analyses) guidelines. A systematic review of the literature to identify studies in the Online MEDLINE (PubMed) and EMBASE reporting on the association of Ollier disease, brain gliomas, and acute myeloid leukemia [17].

This review was performed in accordance with the PRISMA (Preferred Reporting Items for Systematic Reviews and Meta-Analyses) guidelines and has not been registered [17]. Studies in the MEDLINE (PubMed) and EMBASE online electronic databases reporting the association of patients with Ollier disease and brain gliomas and/or acute myeloid leukemia are identified. The search strategy was designed for the identification of English-language articles published until May 2024 using the following relevant keywords, alone or in combination: ‘‘Ollier disease’’, “Ollier syndrome”, ‘‘enchondromatosis’’, ‘‘dyschondroplasia’’, ‘‘glioma’’, ‘‘brain glioma’’, ‘‘cerebral glioma’’, ‘‘glioblastoma’’, ‘‘astrocytoma’’, “oligodendroglioma”, ‘‘myeloid leukemia’’, ‘‘granulocytic leukemia’’, ‘‘myeloblastic leukemia’’ and ‘‘myeloleukemia’’. Additional studies were added based on a review of bibliographies of the identified papers. After duplicate removal, all abstracts were evaluated, and each article of interest was marked for further review. The full text of the marked studies was screened by two authors independently (S.C. and J.B.) and included in this systematic review following inclusion and exclusion criteria, as summarized in Figure 1.

Inclusion criteria encompassed surgical series, reviews, and case reports in the English language, as well as papers written in other languages, including the abstract in English, concerning OD patients with associated brain gliomas and/or acute myeloid leukemia. Patients without a clear diagnosis of OD or with a diagnosis of Mafucci syndrome or enchondromatosis with D-2-hydroxyglutaric aciduria (D-2-HGA) were excluded from the following review.

Reported molecular data concerning Ollier disease, brain gliomas, and acute myeloid leukemia among the enrolled patients were analyzed and discussed.

The consent of our patient was not required since their information was sufficiently anonymized.

## 3. Results

### 3.1. Illustrative Case

**Figure 2:**
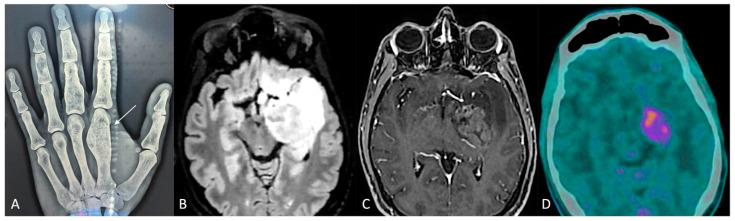
(**A**) Radiograph of the left hand: enchondroma affecting the first metacarpal bone (white arrow). (**B**,**C**) Preoperative brain MRI, axial slices, (**B**) fluid-attenuated inversion recovery (FLAIR), and (**C**) contrast-enhanced sequences. (**D**) PET-CT scan.

A 38-year-old man was affected, since he was an infant, by Ollier disease, mainly localized in both hands (Figure 2A), treated through microsurgical resection of multiple enchondromas of the fingers and whose molecular examination revealed the IDH1 R132H mutation. Four years ago (2020), when he was thirty-five years old, for the onset of complications associated with cytopenia, such as muco-cutaneous bleeding diathesis, mainly in the oral cavity, with fever and weakness, he was referred to the Emergency Department first and then the Hematology Division, where after performing blood tests (White Cells Count 77.000/mmc, Hb 8.5 g/dL, PLT 10.000/mmc, many blasts) and bone marrow aspiration (80% blasts, NPM1 mutant, FLT3 ITD wt, FLT3 TKD mutant, IDH1 p.Arg132His mutant), the diagnosis of acute myeloid leukemia was made. Firstly, the patient underwent frontline induction chemotherapy (“3 + 7” protocol: Idarubicin i.v. in bolus for 3 days and Cytarabine in continuous infusion for 7 days), with the adjunct of Midostaurin, achieving complete remission (<5% blasts on cellular bone marrow aspirate and count recovery with no circulating blasts) and loss of NPM1 and TKD mutations; subsequently, a post-remission three-cycle consolidation chemotherapy was administered, and complete remission, but with IDH1 Arg132His mutation persistence, was confirmed.

Due to the known strict correlation of this mutation in Ollier disease patients with the occurrence of brain gliomas [8], the patient underwent contrast-enhanced magnetic resonance imaging (MRI) of the brain, integrated by perfusion study (2021), which detected a diffuse and infiltrating lesion, hyperintense in T2/FLAIR and hypointense in T1 weighted sequences, without contrast-enhancement nor relative cerebral blood volume (rCBV) values increase, involving the anterior and posterolateral surfaces of the pons, with extension posteriorly toward the cerebellum, caudally toward the medulla oblongata–spinal cord junction, and cranially toward the left cerebral peduncle of the midbrain, hypothalamus, amygdala, temporo-polar and fronto-basal regions, caudate nucleus, and insula. Furthermore, the patient underwent ^11^C-methionine PET/CT with negative results. These findings oriented toward the diagnosis of diffuse infiltrating low-grade brain glioma; nevertheless, a stereotactic cerebral biopsy of the lesion was rejected by the patient, and a “wait and see” strategy through seriate neuroradiological exams and clinical follow-up was adopted.

Six months later, relapsed AML was diagnosed, and the patient underwent allogeneic hemopoietic stem cell transplantation. The following bone marrow aspiration detected complete remission with loss of the IDH1 mutation.

The contrast-enhanced MRI of the brain integrated with perfusion sequences at the last follow-up (December 2023) showed an increase in the size of the known lesion, associated with strong and heterogeneous contrast-enhancement involving the uncus, amygdala, hippocampus, parahippocampal gyrus, and lentiform nucleus, and with increased rCBV values (Figure 2). These findings were in favor of a malignant transformation from low- to high-grade glioma. Therefore, with the assistance of 5-ALA, a neuronavigation system, an intraoperative CT scan, and intraoperative neurophysiological monitoring, the patient underwent microsurgical gross total resection of the contrast-enhancing component of the lesion through pterional craniotomy. The postoperative course was uneventful. The diagnosis was in favor of astrocytoma IDH mutant (IDH1 R132H positive, ATRX mutation, KI67-MIB1 70%, MGMT promoter methylation 55.55%), WHO grade 4. On postoperative day 5, the patient was discharged and addressed to the oncology team for adjuvant therapies.

### 3.2. Literature Search

A detailed and comprehensive systematic literature review disclosed 28 studies which collectively describe 33 patients with OD affected by brain gliomas [3,8,18,19,20,21,22,23,24,25,26,27,28,29,30,31,32,33,34,35,36,37,38,39,40,41,42] and only 1 study reporting one case harboring OD and acute myeloid leukemia [6] (Figure 1). In addition, we reported a single case of a patient with OD who simultaneously experienced both a brain glioma and acute myeloid leukemia.

All patients’ data are summarized in Table 1.

To the best of our knowledge, our patient is the first and unique case of an OD patient who harbored BG and AML and is the second described in the literature affected by AML but the only one with the IDH 1 mutation documented.

Concerning 33 cases of OD patients affected by brain gliomas, most belong to the pre-molecular era; thus, they lack molecular data. In only three cases, molecular analysis was performed, and all of them (100%) exhibited the IDH1 R132H mutation simultaneously in enchondromas and in glioma [8,37,38]; in the unique case of the OD patient affected by acute myeloid leukemia [6], molecular studies were not performed; finally, in our case, the unique patient hosting OD, AML, and BG reported the IDH1 R132H mutation in enchondromas, AML, and brain glioma.

## 4. Discussion

Ollier disease, acute myeloid leukemia, and brain gliomas are three apparently completely different neoplasms in terms of their histopathology, clinic, natural history, and management; OD belongs to the enchondromatosis and is characterized by multiple benign tumors of the cartilaginous tissue, AML is an aggressive tumor of the hematologic series arising from blood cells, and finally, BG is a malignant solid tumor of the central nervous system.

Nevertheless, AML and glioblastoma share several clinical and biochemical features: both malignancies exist predominantly as primary (de novo) forms, with a small percentage of cases occurring as secondary forms, resulting from the progression of precursor diseases, such as myelodysplastic syndromes and low-grade gliomas; but even more important, both primary and secondary forms of both diseases are characterized by common sets of mutations and gene expression abnormalities [43].

Finally, a specific mutation involving the IDH1 gene [8] has been documented in the etiopathogenesis of Ollier disease and is present in most sporadic brain gliomas and acute myeloid leukemia.

The identification of the common molecular pathways and understanding their involvement in the pathogenesis of all three diseases can open the door to the development of new therapeutic targets and/or the application of drugs currently approved for a disease to others sharing common targets.

### 4.1. Diagnostic Molecular Hallmarks

#### 4.1.1. Brain Gliomas

The classification of brain tumors has undergone several changes over the past half-century, mainly due to continued refinements in diagnostic technologies, such as electron microscopy, histochemical stains, immunohistochemistry, in situ hybridization, and molecular genetics and profiling methods, which allowed the identification of new histologic and molecular subtypes. For the past century, the diagnosis was mainly based on histogenesis. In the 2016 WHO classification [44], and even more in the 2021 classification [12], thanks to a better understanding of the genetic basis of tumorigenesis, the role of molecular biology has become decisive, and molecular findings have been incorporated into brain tumor diagnosis and grading. The use of integrated phenotypic and genotypic parameters, resulting from histology and molecular genetics, has provided greater accuracy to the diagnosis, patient management, prognosis, and treatment response.

**Figure 3:**
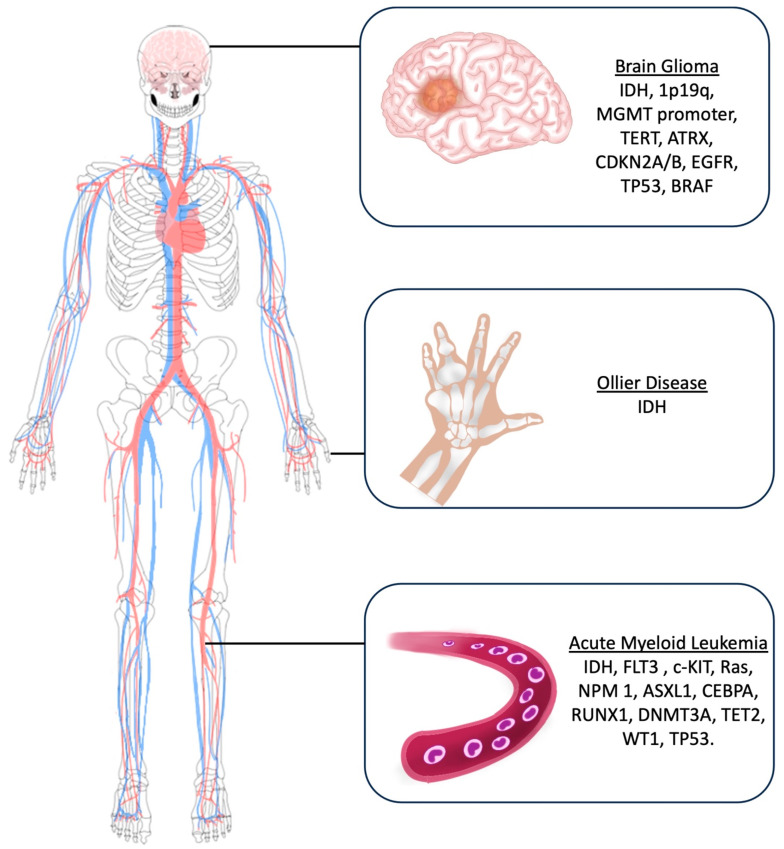
Artistic drawing showing the main molecular markers investigated for brain gliomas, Ollier disease, and acute myeloid leukemia.

The main molecular markers investigated for glioma diagnosis include Isocitrate Dehydrogenase (IDH) mutations, 1p19q deletion, O6-methylguanine-DNA methyltransferase (MGMT) promoter methylation statutes, Telomerase Reverse Transcriptase (TERT) mutations, Alpha-Thalassemia/mental Retardation syndrome X-linked (ATRX), CDKN2A/B deletion, EGFR (Epidermal growth factor receptor) amplification, TP53, and BRAF [14] (Figure 3).

#### 4.1.2. Acute Myeloid Leukemia

Considering the strong impact of new molecular insights regarding AML evolution, progression, and resistance mechanisms on the diagnosis, classification, and prognosis of patients with AML [15,16,45], the updated [15] and the newest [16] AML classification system emphasizes the integration of molecular analysis into daily practice in AML.

In this setting, main molecular markers investigated in AML patients include IDH, FLT3 (Fms-Like Tyrosine Kinase 3), c-KIT, Ras, NPM1 (Nucleophosmin 1), ASXL1 (Additional sex comb-like 1), CEBPA (CCAAT Enhancer Binding Protein α), RUNX1 (Run-related transcription factor), DNMT3A (DNA methyltransferase 3A), TET2 (Ten-eleven translocation), WT1 (Wilm’s tumor), and TP53 (Tumor Protein) (Figure 3).

#### 4.1.3. Ollier Disease

While the underlying cause of Ollier disease is unknown, and most of the reported cases belong to the pre-molecular era, the current literature postulates that the etiology of Ollier disease is related to an early post-zygotic gain-of-function sporadic and non-heritable mutation in the isocitrate dehydrogenases 1 and 2 (IDH1 and IDH2) [46,47,48,49]. Therefore, molecular studies to identify IDH mutations are suggested during the diagnostic process of OD (Figure 3).

### 4.2. Isocitrate Dehydrogenase (IDH): The Common Denominator

Two IDH variants, namely IDH1 and IDH2, are involved in tumorigenesis. Both genes encode for two enzymes of the Krebs cycle, which catalyze the conversion of isocitrate to alpha-ketoglutarate. IDH 1 mutations are heterozygous and involve an amino acid substitution (glycine to arginine) in the active site of the enzyme in codon 132 (R132H). This mutation results in the abnormal production of 2-hydroxyglutarate, an oncometabolite which causes histone and DNA methylation, hence promoting tumorigenesis.

IDH2 mutations involve codon 172, resulting in 2-hydroxyglutaric aciduria, accounting for seizures, hypotonia, and progressive cerebral damage.

Major advances in cancer genetics over the past decade have documented the involvement of IDH mutations in a variety of human malignancies, including gliomas, acute myeloid leukemia, chondrosarcoma, cholangiocarcinoma, and thyroid carcinoma, but also in prostate adenocarcinoma and non-small cell lung cancer [50,51,52]. In particular, IDH gene mutations are the most common genetic alterations in brain gliomas [51] and in most acute myeloid leukemias [53,54,55].

Among the conventional molecular markers commonly investigated for diagnosis and management of each one of the three considered pathologies—OD, AML, and BG—IDH1 R132H represents the only genetic anomaly shared by all three diseases.

IDH1 mutations are present in both precursors of AML and glioblastoma, i.e., myelodysplastic syndromes (MDS) (5%) and diffuse anaplastic astrocytoma (>80%), with different impacts on their malignant transformation rate; this mutation accounts for malignant transformation in 50% of cases of MDS, while it is not associated with the rate nor the time to malignant transformation of low-grade gliomas [43].

While the IDH1 mutation is separately detected in more than 80% of gliomas, 7–14% of cytogenetically normal AML, and 90% of enchondromatosis, with the most frequent mutation type represented by R132H [50,51], from our literature review, this last one is simultaneously present in enchondromas, brain gliomas, and acute myeloid leukemia in all cases where molecular analysis was performed, in detail, in the three cases of OD with BG, and in our case where all three diseases coexisted. These findings might suggest that the IDH mutation may not be simply the initial etiopathogenetic factor of cartilaginous tumors but rather an intrinsic genetic link among multiple apparently different neoplastic conditions in the setting of somatic mosaicism.

#### 4.2.1. IDH and Gliomas

Since their first identification in 2008 in a subgroup of diffusely infiltrating gliomas, the isocitrate dehydrogenase mutations have played a leading role in the diagnostic and therapeutic approach to gliomas, initially with the inclusion of the IDH-mutation status into the 2016 WHO Classification of the tumors of the CNS [44], and subsequently accounting for the definition of a distinct family of tumors ranging from low-grade to high-grade in the most recent 2021 Classification [12], including astrocytoma and oligodendroglioma. Different IDH status is associated with different clinical behaviors and responses to treatment.

While mutation of both IDH1 and IDH2 isoforms are observed in a range of cancers [56], IDH1 R132H represents the main mutation (90%) in sporadic IDH mutant gliomas [57] and is associated with more favorable outcomes. Indeed, the most aggressive glioma type, namely glioblastoma, which is a grade 4 malignancy, is the IDH wild-type.

#### 4.2.2. IDH and AML

The IDH mutations in AML were first described in 2008 in concert with sequencing of the first AML genome [58]. It is now recognized that mutations of this gene occur in up to 20% of adult patients with AML [59,60] and 30% of pediatric AML [61], and drive leukemogenesis [54].

The impact of IDH mutations on AML prognosis remains controversial and context-dependent [50,53].

#### 4.2.3. IDH and Ollier Disease

While IDH mutations are reported in over 80% of OD patients [48,62], their role in the etiopathogenesis of OD and in the risk of malignant transformation is still a matter of debate.

### 4.3. Current Therapeutic Potential of IDH Mutations: Targeting IDH

#### 4.3.1. IDH Inhibitors in AML

Since the first discovery of cancer-associated IDH1 and IDH2 mutations, IDH-inhibitor drugs are available for patients with AML harboring these mutations, both in newly diagnosed patients not eligible for intensive therapy in which Ivosidenib (IDH1 inhibitor) accounts for a rate of 42% of complete remission/partial hematologic recovery and overall survival of 12% and in those in the relapsed/refractory setting in which Ivosidenib and Enasidenib account for a rate of 30% of complete remission/partial hematologic recovery and a median overall survival of 9 months [10,55,63]. The rate of complete remission/partial hematologic recovery exceeds 60% when IDH inhibitors are associated with other treatments, like azacytidine and the standard 7 + 3 chemotherapy approach [10,55].

Second-generation and “pan” IDH inhibitors are currently under investigation.

#### 4.3.2. IDH Inhibitors in Gliomas

The central role of IDH mutations in glioma genesis is highlighted by ubiquitous expression throughout the tumor and persistence of the mutation during disease in most cases [13].

Treatment with a mutant IDH-specific inhibitor that blocks D-2-hydroxyglutarate production impairs glioma growth in preclinical studies. A phase I study evaluating Ivosidenib reported a prolonged stable disease and reduced growth of the non-enhancing tumor [64]. The results from the INDIGO trial [65] on the IDH inhibitor, Vorasidenib showed a significant improvement in progression-free survival (PFS) and delayed time to next treatment among patients harboring grade 2 IDH mutant gliomas per WHO 2016 Classification without contrast-enhancement on MRI and not previously treated with radio- or chemotherapy, whereas gliomas showing contrast-enhancement on MRI experimented much less benefit in terms of radiological response to this drug [66].

Current approaches for each one of the three different diseases include exeresis of enchondromas for OD, maximal safe tumor resection followed by radio-, with or without chemotherapy according to the WHO tumor grade for IDH mutant gliomas [13], and induction therapy followed by post-remission therapy administration, represented by chemotherapy and/or allogenic stem cell transplantation for AML [10]. To the best of our knowledge, our reported case represents the first one hosting all these three conditions, which exhibit the same gene mutation; therefore, guidelines of management do not exist, and the three pathologies have been separately treated. Nevertheless, an intrinsic genetic link among multiple neoplastic conditions in the setting of somatic mosaicism might be hypothesized.

Some questions spontaneously arise: does the persistence/loss of IDH mutation after allogenic stem cell transplantation affect the occurrence risk of brain glioma IDH mutant in AML IDH-mutant patients? Does the treatment with IDH inhibitors for AML affect the occurrence risk of brain glioma IDH mutant? What is the impact of IDH inhibitor treatment on the risk of malignant transformation of enchondromas?

Low-grade gliomas (LGGs) are slow-growing, infiltrating, and often asymptomatic groups of tumors that exhibit peculiar molecular, clinical, and histological features and whose natural history, ranging from indolent to biologically aggressive behavior with an exponential, not linear course, is not fully understood. Although the “wait and see” approach still represents a strategy of treatment in asymptomatic patients in some institutions, long-term follow-up data showed that these lesions unpredictably but invariably undergo malignant transformation [67], inexorably leading to death [68,69]. Even when seriate imaging studies show unchanged findings, not-detectable background molecular mutations occur. Therefore, in light of this data and considering that the overall survival is worst in secondary high-grade gliomas (median 2 years after progression) [70,71] compared to that one of unchanged low-grade histology at recurrence [72], most authors suggest an early diagnosis and preventive maximal safe tumor resection improve the outcome of LGG patients [73,74]. The rate of malignant transformation, as well as the rate of progression-free survival, reported in the literature, are extremely heterogeneous, and several pre- and postoperative factors [75,76] have been considered; in regard to the 10-year malignant progression-free survival (MPFS), the rates range from 22.4% to 75% [69,75,77,78,79]. Despite the discovery and introduction of several molecular markers that represent key elements in stratifying LGGs, currently, an objective and prognostic classification is lacking, thus rendering the time of therapeutic intervention arbitrary [80].

Due to the strong association of brain glioma with Ollier’s disease, it is justified to offer a treatment with vorasidenib to a patient with a diagnosis of OD IDH R132H mutant, with or without cured AML IDH 132H mutant and with neuroradiological findings suggestive of diffuse low-grade glioma but who refused biopsy of the cerebral lesion?

Further studies and larger clinical series are needed to confirm our results and hypothesis and to answer several questions.

### 4.4. Advantages and Limits

The first limitation of this study is its retrospective nature. Another limit is the small sample and the heterogeneity of the available data.

The purpose of the study is to emphasize the etiopathogenetic role of IDH mutation and its potential therapeutic role simultaneously for Ollier Disease, brain gliomas, and acute myeloid leukemia. Clinical trials are needed to confirm our results and hypothesis.

## 5. Conclusions and Future Perspective

The IDH1-R132H gene mutation is the etiopathogenetic common denominator among three apparently different neoplasms, namely Ollier disease, acute myeloid leukemia, and brain glioma, with different nature, biology, and clinic history, and which require different treatments but which can coexist in the same patients.

The adoption of mutant-specific IDH1 inhibitor molecules in patients affected by Ollier disease, acute myeloid leukemia, and brain gliomas could represent a potential therapeutic solution in the era of targeted therapy. Further studies, including larger case series and complete molecular data, are needed to confirm our hypothesis.

## Figures and Tables

**Table 1:** Demographic, pathological, treatment, and outcome data of patients with Ollier disease and affected by brain gliomas and/or acute myeloid leukemia.

OD + BG								
Becker et al. 1979 [18]	26/NA	Oligoastrocytoma (Grade II)	NA	NA	NA	NA	NA	NA
Rawlings et al. 1987 [19]	29/M	Anaplastic astrocytoma (Grade III)	NA	NA	Biopsy	Radiotherapy	NA	NA
Schwartz et al. 1987 [20]	38/M	Malignant astrocytoma (Grade III)	NA	NA	NA	NA	NA	Exitus
Mellon et al. 1988 [21]	34/M	Astrocytoma (Grade II)	NA	NA	Craniotomy	NA	NA	NA
Patt et al. 1990 [22]	24/M	Astrocytoma (Grade II)	NA	NA	Biopsy	NA	NA	NA
Bendel et al. 1991 [23]	29/F	High grade astrocytoma (Grade III)	NA	NA	NA	NA	NA	NA
Chang et al. 1994 [24]	23/M	Anaplastic astrocytoma (Grade III)	NA	NA	Biopsy	WBRT	Absent (3y)	Alive (3y)
Chang et al. 1994 [24]	25/M	Oligodendroglioma (NA)	NA	NA	Craniotomy (STR)	Radiotherapy	Absent (8m)	Alive (8m)
Chang et al. 1994 [24]	46/M	Oligoastrocytoma (NA)	NA	NA	Biopsy	Radiotherapy	Absent (4m)	Alive (4m)
Hofman et al. 1998 [25]	28/M	Astrocytoma (Grade II)	NA	NA	Biopsy	Radiotherapy	Absent (1y)	Alive (1y)
Balcer et al. 1999 [26]	23/F	Astrocytoma (NA)	NA	NA	None	Radiotherapy	NA	NA
Frappaz et al. 1999 [27]	16/M	Astrocytoma (NA)	NA	NA	None	Radiotherapy	Absent (7m)	Alive (7m)
Van Nielen et al. 1999 [28]	28/M	Astrocytoma (Grade II)	NA	NA	Biopsy	Radiotherapy	NA	NA
Simsek et al. 2002 [29]	7/F	Astrocytoma (Grade II)	NA	NA	NA	NA	NA	NA
Mahafza et al. 2004 [30]	21/F	Astrocytoma (Grade II)	NA	NA	Biopsy	NA	NA	NA
Koc et al. 2006 [31]	28/F	Anaplastic astrocytoma (Grade III)	NA	NA	Craniotomy	Radiotherapy at recurrence	Recurrence (6y)—Reoperation	Alive (10y)
Ranger et al. 2009 [32]	6/F	Glioblastoma (Grade IV)	NA	NA	Biopsy	Radiotherapy + Chemotherapy	Progression	Exitus (11m)
Walid et al. 2008 [33]	14/M	Anaplastic astrocytoma (Grade III)	NA	NA	NA	NA	NA	NA
Hori et al. 2010 [34]	19/M	Anaplastic astrocytoma (Grade III)	NA	NA	Craniotomy	Radiotherapy + TMZ	Stable	Alive (NA)
Bathla et al. 2012 [35]	16/M	Astrocytoma (Grade II)	IDH1 R132H	NA	Biopsy	Chemotherapy Craniotomy at progression	Progression (3y), reoperation—anaplastic astrocytoma (grade III),	Exitus (8y)
Pearce et al. 2012 [36]	19/M	Oligoastrocytoma (Grade II)	No mutation	NA	Biopsy	None	Progression (9m)—Biopsy and craniotomy	NA
Gajavelli et al. 2016 [3]	55/F	Anaplastic astrocytoma (Grade III)	IDH1 R132H	NA	Craniotomy (STR)	None	Stable (3y)	Alive (3y)
Bonnet et al. 2016 [37]	28/F	Oligoastrocytoma (Grade II)	IDH1 R132H	NA	Biopsy	NA	NA	Alive (2.5y)
Bonnet et al. 2016 [37]	26/M	NA	NA	NA	Biopsy	NA	NA	Alive (1y)
Bonnet et al. 2016 [37]	20/F	Oligodendroglioma (Grade II)	IDH1 R132H	NA	Biopsy	NA	NA	Alive (4y)
Bonnet et al. 2016 [37]	31/M	Glioblastoma (Grade IV)	IDH1 R132H	NA	Biopsy	NA	NA	Alive (9m)
Bonnet et al. 2016 [37]	31/F	Oligoastrocytoma (Grade II)	IDH1 R132H	IDH1 R132H	Biopsy	NA	NA	Alive (1.5y)
Achiha et al. 2017 [38]	32/M	Oligodendroglioma (NA)	IDH1 R132H	IDH1 R132H	NA	NA	NA	NA
Al Rumeh et al. 2020 [39]	23/F	Diffuse astrocytoma (Grade II)	IDH1 R132C	NA	Craniotomy	NA	NA	NA
Karabulut et al. 2021 [40]	23/F	Diffuse midline glioma (NA)	NA	NA	Observation	Radiotherapy + Chemotherapy	Stable	Alive (NA)
Gregory et al. 2021 [41]	14/F	Astrocytoma (Grade II)	NA	NA	Craniotomy	NA	NA	NA
Corvino et al. 2022 [8]	33/M	Diffuse astrocytoma (Grade II)	IDH1 R132H	IDH1 R132H	Biopsy	Radiotherapy + TMZ	Progression (48m) biopsy, operation + RT	Alive (6y)
Ikeda et al. 2023 [42]	24/F	Astrocytoma Oligodendroglioma	IDH1 R132H	NA	Craniotomy (GTR)	Chemotherapy	NA	Alive (21m)
OD + AML								
White et al. 2008 [6]	7/M	AML, FAB subtype M1	NA	NA	Chemotherapy	Bone Marrow Transplant	No	Complete remission(19 m)
OD + BG + AML								
Present case	38/M	Enchondromatosis OD	IDH1 R132H	Surgery	No	No		Stable
		Astrocytoma grade 4	IDH1 R132H	Surgery	From low-grade at MRI	RT + CHT		Stable at 3 months
		Acute Myeloid Leukemia	IDH1 R132H	Induction chemotherapy follower by ATS	Relapsed AML with IDH1 R132 H mutation persistence		Allogeneic hemopoietic stem cell transplantation	Complete remission with IDH1 R132 H mutation lost

NA: Not available; M: Male; F: Female.

## References

[1] D’Angelo L., Massimi L., Narducci A., Di Rocco C. (2009). Ollier disease. Childs Nerv. Syst..

[2] Silve C., Jüppner H. (2006). Ollier disease. Orphanet J. Rare Dis..

[3] Gajavelli S., Nakhla J., Nasser R., Yassari R., Weidenheim K.M., Graber J. (2016). Ollier disease with anaplastic astrocytoma: A review of the literature and a unique case. Surg. Neurol. Int..

[4] Verdegaal S.H., Bovée J.V., Pansuriya T.C., Grimer R.J., Ozger H., Jutte P.C., San Julian M., Biau D.J., van der Geest I.C., Leithner A. (2011). Incidence, predictive factors, and prognosis of chondrosarcoma in patients with Ollier disease and Maffucci syndrome: An international multicenter study of 161 patients. Oncologist.

[5] El Abiad J.M., Robbins S.M., Cohen B., Levin A.S., Valle D.L., Morris C.D., de Macena Sobreira N.L. (2020). Natural history of Ollier disease and Maffucci syndrome: Patient survey and review of clinical literature. Am. J. Med. Genet. Part A.

[6] White M.S., Martin P.L., McLean T.W. (2008). Acute myelogenous leukemia associated with Ollier disease. Pediatr. Blood Cancer.

[7] Tamimi H.K., Bolen J.W. (1984). Enchondromatosis (Ollier’s disease) and ovarian juvenile granulosa cell tumor. Cancer.

[8] Corvino S., Mariniello G., Corazzelli G., Franca R.A., Del Basso De Caro M., Della Monica R., Chiariotti L., Maiuri F. (2022). Brain Gliomas and Ollier Disease: Molecular Findings as Predictive Risk Factors?. Cancers.

[9] DiNardo C.D., Erba H.P., Freeman S.D., Wei A.H. (2023). Acute myeloid leukaemia. Lancet.

[10] Shimony S., Stahl M., Stone R.M. (2023). Acute myeloid leukemia: 2023 update on diagnosis, risk-stratification, and management. Am. J. Hematol..

[11] SEER Data Base: Cancer Stat Facts: Leukemia—Acute Myeloid Leukemia (AML). https://seer.cancer.gov/statfacts/html/amyl.html.

[12] Louis D.N., Perry A., Wesseling P., Brat D.J., Cree I.A., Figarella-Branger D., Hawkins C., Ng H.K., Pfister S.M., Reifenberger G. (2021). The 2021 WHO Classification of Tumors of the Central Nervous System: A summary. Neuro-Oncology.

[13] Miller J.J., Gonzalez Castro L.N., McBrayer S., Weller M., Cloughesy T., Portnow J., Andronesi O., Barnholtz-Sloan J.S., Baumert B.G., Berger M.S. (2023). Isocitrate dehydrogenase (IDH) mutant gliomas: A Society for Neuro-Oncology (SNO) consensus review on diagnosis, management, and future directions. Neuro-Oncology.

[14] Sahm F., Brandner S., Bertero L., Capper D., French P.J., Figarella-Branger D., Giangaspero F., Haberler C., Hegi M.E., Kristensen B.W. (2023). Molecular diagnostic tools for the World Health Organization (WHO) 2021 classification of gliomas, glioneuronal and neuronal tumors; an EANO guideline. Neuro-Oncology.

[15] Khoury J.D., Solary E., Abla O., Akkari Y., Alaggio R., Apperley J.F., Bejar R., Berti E., Busque L., Chan J.K.C. (2022). The 5th edition of the World Health Organization Classification of Haematolymphoid Tumours: Myeloid and Histiocytic/Dendritic Neoplasms. Leukemia.

[16] Arber D.A., Orazi A., Hasserjian R.P., Borowitz M.J., Calvo K.R., Kvasnicka H.M., Wang S.A., Bagg A., Barbui T., Branford S. (2022). International Consensus Classification of Myeloid Neoplasms and Acute Leukemias: Integrating morphologic, clinical, and genomic data. Blood.

[17] Page M.J., McKenzie J.E., Bossuyt P.M., Boutron I., Hoffmann T.C., Mulrow C.D., Shamseer L., Tetzlaff J.M., Akl E.A., Brennan S.E. (2021). The PRISMA 2020 statement: An updated guideline for reporting systematic reviews. BMJ.

[18] Becker W., Thron A. (1979). Dyschondroplasia with glioma of the brain. Third histologically verified case. Arch. Orthop. Trauma Surg..

[19] Rawlings C.E., Bullard D.E., Burger P.C., Friedman A.H. (1987). A case of Ollier’s disease associated with two intracranial gliomas. Neurosurgery.

[20] Schwartz H.S., Zimmerman N.B., Simon M.A., Wroble R.R., Millar E.A., Bonfiglio M. (1987). The malignant potential of enchondromatosis. J. Bone Jt. Surg..

[21] Mellon C.D., Carter J.E., Owen D.B. (1988). Ollier’s disease and Maffucci’s syndrome: Distinct entities or a continuum. J. Neurol..

[22] Patt S., Weigel K., Mayer H.M. (1990). A case of dyschondroplasia associated with brain stem glioma: Diagnosis by stereotactic biopsy. Neurosurgery.

[23] Bendel C.J., Gelmers H.J. (1991). Multiple enchondromatosis (Ollier’s disease) complicated by malignant astrocytoma. Eur. J. Radiol..

[24] Chang S., Prados M.D. (1994). Identical twins with Ollier’s disease and intracranial gliomas: Case report. Neurosurgery.

[25] Hofman S., Heeg M., Klein J.P., Krikke A.P. (1998). Simultaneous occurrence of a supra- and an infratentorial glioma in a patient with Ollier’s disease: More evidence for non-mesodermal tumor predisposition in multiple enchondromatosis. Skelet. Radiol..

[26] Balcer L.J., Galetta S.L., Cornblath W.T., Liu G.T. (1999). Neuro-ophthalmologic manifestations of Maffucci’s syndrome and Ollier’s disease. J. Neuro-Ophthalmol..

[27] Frappaz D., Ricci A.C., Kohler R., Bret P., Mottolese C. (1999). Diffuse brain stem tumor in an adolescent with multiple enchondromatosis (Ollier’s disease). Childs Nerv. Syst..

[28] van Nielen K.M., de Jong B.M. (1999). A case of Ollier’s disease associated with two intracerebral low-grade gliomas. Clin. Neurol. Neurosurg..

[29] Simsek S., Seckin H., Belin D. (2002). Ollier’s disease with intracranial glioma. Türk Nörosirürji Derg..

[30] Mahafza W.S. (2004). Multiple enchondromatosis Ollier’s disease with two primary brain tumors. Saudi Med. J..

[31] Koc F. (2006). Ollier Disease Anaplastic Mixed Oligoastrocytoma: A Rare Association with Brain Tumors. Neurosurg. Q..

[32] Ranger A., Szymczak A., Hammond R.R., Zelcer S. (2009). Pediatric thalamic glioblastoma associated with Ollier disease (multiple enchondromatosis): A rare case of concurrence. J. Neurosurg. Pediatr..

[33] Walid M.S., Troup E.C. (2008). Cerebellar anaplastic astrocytoma in a teenager with Ollier Disease. J. Neuro-Oncol..

[34] Hori K., Matsumine A., Niimi R., Maeda M., Uchida K., Nakamura T., Sudo A. (2010). Diffuse gliomas in an adolescent with multiple enchondromatosis (Ollier’s disease). Oncol. Lett..

[35] Bathla G., Gupta S., Ong C.K. (2012). Multifocal intracranial astrocytoma in a pediatric patient with Ollier disease. Indian J. Radiol. Imaging.

[36] Pearce P., Robertson T., Ortiz-Gomez J.D., Rajah T., Tollesson G. (2012). Multifocal supratentorial diffuse glioma in a young patient with Ollier disease. J. Clin. Neurosci..

[37] Bonnet C., Thomas L., Psimaras D., Bielle F., Vauléon E., Loiseau H., Cartalat-Carel S., Meyronet D., Dehais C., Honnorat J. (2016). Characteristics of gliomas in patients with somatic IDH mosaicism. Acta Neuropathol. Commun..

[38] Achiha T., Arita H., Kagawa N., Murase T., Ikeda J.I., Morii E., Kanemura Y., Fujimoto Y., Kishima H. (2018). Enchondromatosis-associated oligodendroglioma: Case report and literature review. Brain Tumor Pathol..

[39] Al Rumeh A.S. (2020). Diffuse astrocytoma and Ollier’s Disease. J. Neurol. Res..

[40] Karabulut A.K., Türk S., Tamsel İ., Kim J., Argın M. (2021). Diffuse midline glioma in Ollier disease: A case report and a brief review of the literature. Radiol. Case Rep..

[41] Gregory T.A., Taylor L.P. (2021). Teaching NeuroImage: Histopathologically Confirmed Intracranial Enchondroma/Low-Grade Chondrosarcoma and *IDH1*-Mutated Diffuse Glioma in Ollier Disease. Neurology.

[42] Ikeda H., Yamaguchi S., Ishi Y., Wakabayashi K., Shimizu A., Kanno-Okada H., Endo T., Ota M., Okamoto M., Motegi H. (2023). Supratentorial multifocal gliomas associated with Ollier disease harboring IDH1 R132H mutation: A case report. Neuropathology.

[43] Goethe E., Carter B.Z., Rao G., Pemmaraju N. (2018). Glioblastoma and acute myeloid leukemia: Malignancies with striking similarities. J. Neuro-Oncol..

[44] Louis D.N., Ohgaki H., Wiestler O.D., Cavenee W.K., World Health Organization (2016). WHO Classification of Tumours of the Central Nervous System.

[45] Papaemmanuil E., Gerstung M., Bullinger L., Gaidzik V.I., Paschka P., Roberts N.D., Potter N.E., Heuser M., Thol F., Bolli N. (2016). Genomic Classification and Prognosis in Acute Myeloid Leukemia. N. Engl. J. Med..

[46] Amary M.F., Bacsi K., Maggiani F., Damato S., Halai D., Berisha F., Pollock R., O’Donnell P., Grigoriadis A., Diss T. (2011). IDH1 and IDH2 mutations are frequent events in central chondrosarcoma and central and periosteal chondromas but not in other mesenchymal tumours. J. Pathol..

[47] Amary M.F., Damato S., Halai D., Eskandarpour M., Berisha F., Bonar F., McCarthy S., Fantin V.R., Straley K.S., Lobo S. (2011). Ollier disease and Maffucci syndrome are caused by somatic mosaic mutations of IDH1 and IDH2. Nat. Genet..

[48] Pansuriya T.C., van Eijk R., d’Adamo P., van Ruler M.A., Kuijjer M.L., Oosting J., Cleton-Jansen A.M., van Oosterwijk J.G., Verbeke S.L., Meijer D. (2011). Somatic mosaic IDH1 and IDH2 mutations are associated with enchondroma and spindle cell hemangioma in Ollier disease and Maffucci syndrome. Nat. Genet..

[49] Tan C.L., Vellayappan B., Wu B., Yeo T.T., McLendon R.E. (2018). Molecular profiling of different glioma specimens from an Ollier disease patient suggests a multifocal disease process in the setting of IDH mosaicism. Brain Tumor Pathol..

[50] Bruce-Brand C., Govender D. (2020). Gene of the month: *IDH1*. J. Clin. Pathol..

[51] Dang L., Jin S., Su S.M. (2010). IDH mutations in glioma and acute myeloid leukemia. Trends Mol. Med..

[52] Dang L., Yen K., Attar E.C. (2016). IDH mutations in cancer and progress toward development of targeted therapeutics. Ann. Oncol..

[53] Kunadt D., Stasik S., Metzeler K.H., Röllig C., Schliemann C., Greif P.A., Spiekermann K., Rothenberg-Thurley M., Krug U., Braess J. (2022). Impact of IDH1 and IDH2 mutational subgroups in AML patients after allogeneic stem cell transplantation. J. Hematol. Oncol..

[54] Steinhäuser S., Silva P., Lenk L., Beder T., Hartmann A., Hänzelmann S., Fransecky L., Neumann M., Bastian L., Lipinski S. (2023). Isocitrate dehydrogenase 1 mutation drives leukemogenesis by PDGFRA activation due to insulator disruption in acute myeloid leukemia (AML). Leukemia.

[55] Messina M., Piciocchi A., Ottone T., Paolini S., Papayannidis C., Lessi F., Fracchiolla N.S., Forghieri F., Candoni A., Mengarelli A. (2022). Prevalence and Prognostic Role of IDH Mutations in Acute Myeloid Leukemia: Results of the GIMEMA AML1516 Protocol. Cancers.

[56] Pirozzi C.J., Yan H. (2021). The implications of IDH mutations for cancer development and therapy. Nat. Rev. Clin. Oncol..

[57] Yan H., Parsons D.W., Jin G., McLendon R., Rasheed B.A., Yuan W., Kos I., Batinic-Haberle I., Jones S., Riggins G.J. (2009). IDH1 and IDH2 mutations in gliomas. N. Engl. J. Med..

[58] Mardis E.R., Ding L., Dooling D.J., Larson D.E., McLellan M.D., Chen K., Koboldt D.C., Fulton R.S., Delehaunty K.D., McGrath S.D. (2009). Recurring mutations found by sequencing an acute myeloid leukemia genome. N. Engl. J. Med..

[59] Figueroa M.E., Abdel-Wahab O., Lu C., Ward P.S., Patel J., Shih A., Li Y., Bhagwat N., Vasanthakumar A., Fernandez H.F. (2010). Leukemic IDH1 and IDH2 mutations result in a hypermethylation phenotype, disrupt TET2 function, and impair hematopoietic differentiation. Cancer Cell.

[60] Paschka P., Schlenk R.F., Gaidzik V.I., Habdank M., Krönke J., Bullinger L., Späth D., Kayser S., Zucknick M., Götze K. (2010). IDH1 and IDH2 mutations are frequent genetic alterations in acute myeloid leukemia and confer adverse prognosis in cytogenetically normal acute myeloid leukemia with NPM1 mutation without FLT3 internal tandem duplication. J. Clin. Oncol..

[61] Damm F., Thol F., Hollink I., Zimmermann M., Reinhardt K., van den Heuvel-Eibrink M.M., Zwaan C.M., de Haas V., Creutzig U., Klusmann J.H. (2011). Prevalence and prognostic value of IDH1 and IDH2 mutations in childhood AML: A study of the AML-BFM and DCOG study groups. Leukemia.

[62] Chen C., Li J., Jiang T., Tang J., Zhang Z., Luo Y., Wang X., Sun K., Jiang Z., Zhou J. (2022). *IDH* Mutations Are Potentially the Intrinsic Genetic Link among the Multiple Neoplastic Lesions in Ollier Disease and Maffucci Syndrome: A Clinicopathologic Analysis from a Single Institute in Shanghai, China. Diagnostics.

[63] Okoye-Okafor U.C., Bartholdy B., Cartier J., Gao E.N., Pietrak B., Rendina A.R., Rominger C., Quinn C., Smallwood A., Wiggall K.J. (2015). New IDH1 mutant inhibitors for treatment of acute myeloid leukemia. Nat. Chem. Biol..

[64] Mellinghoff I.K., Ellingson B.M., Touat M., Maher E., De La Fuente M.I., Holdhoff M., Cote G.M., Burris H., Janku F., Young R.J. (2020). Ivosidenib in Isocitrate Dehydrogenase 1—Mutated Advanced Glioma. J. Clin. Oncol..

[65] Mellinghoff I.K., van den Bent M.J., Blumenthal D.T., Touat M., Peters K.B., Clarke J., Mendez J., Yust-Katz S., Welsh L., Mason W.P. (2023). Vorasidenib in IDH1- or IDH2-Mutant Low-Grade Glioma. N. Engl. J. Med..

[66] Mellinghoff I.K., Lu M., Wen P.Y., Taylor J.W., Maher E.A., Arrillaga-Romany I., Peters K.B., Ellingson B.M., Rosenblum M.K., Chun S. (2023). Vorasidenib and ivosidenib in IDH1-mutant low-grade glioma: A randomized, perioperative phase 1 trial. Nat. Med..

[67] Claus E.B., Walsh K.M., Wiencke J.K., Molinaro A.M., Wiemels J.L., Schildkraut J.M., Bondy M.L., Berger M., Jenkins R., Wrensch M. (2015). Survival and low-grade glioma: The emergence of genetic information. Neurosurg. Focus.

[68] Jansen E., Hamisch C., Ruess D., Heiland D.H., Goldbrunner R., Ruge M.I., Schnell O., Grau S.J. (2019). Observation after surgery for low grade glioma: Long-term outcome in the light of the 2016 WHO classification. J. Neuro-Oncol..

[69] Thon N., Eigenbrod S., Kreth S., Lutz J., Tonn J.C., Kretzschmar H., Peraud A., Kreth F.W. (2012). *IDH1* mutations in grade II astrocytomas are associated with unfavorable progression-free survival and prolonged postrecurrence survival. Cancer.

[70] Capelle L., Fontaine D., Mandonnet E., Taillandier L., Golmard J.L., Bauchet L., Pallud J., Peruzzi P., Baron M.H., Kujas M. (2013). Spontaneous and therapeutic prognostic factors in adult hemispheric World Health Organization Grade II gliomas: A series of 1097 cases: Clinical article. J. Neurosurg..

[71] Tom M.C., Park D.Y.J., Yang K., Leyrer C.M., Wei W., Jia X., Varra V., Yu J.S., Chao S.T., Balagamwala E.H. (2019). Malignant Transformation of Molecularly Classified Adult Low-Grade Glioma. Int. J. Radiat. Oncol. Biol. Phys..

[72] Narang A.K., Chaichana K.L., Weingart J.D., Redmond K.J., Lim M., Olivi A., Quinones-Hinojosa A., Kleinberg L.R. (2017). Progressive Low-Grade Glioma: Assessment of Prognostic Importance of Histologic Reassessment and MRI Findings. World Neurosurg..

[73] Lima G.L.d.O., Zanello M., Mandonnet E., Taillandier L., Pallud J., Duffau H. (2016). Incidental diffuse low-grade gliomas: From early detection to preventive neuro-oncological surgery. Neurosurg. Rev..

[74] Duffau H. (2016). Long-term outcomes after supratotal resection of diffuse low-grade gliomas: A consecutive series with 11-year follow-up. Acta Neurochir..

[75] Nakasu S., Nakasu Y. (2022). Malignant Progression of Diffuse Low-grade Gliomas: A Systematic Review and Meta-analysis on Incidence and Related Factors. Neurol. Med. Chir..

[76] Satar Z., Hotton G., Samandouras G. (2021). Systematic review-Time to malignant transformation in low-grade gliomas: Predicting a catastrophic event with clinical, neuroimaging, and molecular markers. Neuro-Oncol. Adv..

[77] Cesselli D., Ius T., Isola M., Del Ben F., Da Col G., Bulfoni M., Turetta M., Pegolo E., Marzinotto S., Scott C.A. (2019). Application of an Artificial Intelligence Algorithm to Prognostically Stratify Grade II Gliomas. Cancers.

[78] Chaichana K.L., McGirt M.J., Laterra J., Olivi A., Quiñones-Hinojosa A. (2010). Recurrence and malignant degeneration after resection of adult hemispheric low-grade gliomas. J. Neurosurg..

[79] van den Bent M.J., Afra D., de Witte O., Ben Hassel M., Schraub S., Hoang-Xuan K., Malmström P.O., Collette L., Piérart M., Mirimanoff R. (2005). Long-term efficacy of early versus delayed radiotherapy for low-grade astrocytoma and oligodendroglioma in adults: The EORTC 22845 randomised trial. Lancet.

[80] Veeravagu A., Jiang B., Ludwig C., Chang S.D., Black K.L., Patil C.G. (2013). Biopsy versus resection for the management of low-grade gliomas. Cochrane Database Syst. Rev..

